# Identifying and predicting dietary patterns in the Dutch population using machine learning

**DOI:** 10.1007/s00394-025-03817-4

**Published:** 2025-10-23

**Authors:** Marlijn L. van Houwelingen, Yinjie Zhu

**Affiliations:** 1 Consumption and Healthy Lifestyles Chair Group, Wageningen University & Research, Hollandseweg 1, 6706 KN Wageningen, The Netherlands; 2 National Institute for Public Health and the Environment, Antonie Van Leeuwenhoeklaan 9, 3721 MA Bilthoven, The Netherlands

**Keywords:** Classification, Cluster analysis, Dietary patterns, Machine learning

## Abstract

**Purpose:**

Nutritional epidemiological research is shifting its focus from individual nutrients to dietary patterns, which challenges traditional statistical methods. Here, we aim to apply various machine learning algorithms to identify and predict dietary patterns in the Dutch population.

**Methods:**

Data on food consumption, sociodemographic and lifestyle factors from 867 males and 866 females participating in the Dutch National Food Consumption Survey (DNFCS) were analysed. K-means, K-medoids, and hierarchical clustering were compared to identify dietary patterns by sex. Six classifiers (naïve Bayes, K-nearest neighbours, decision tree, random forest, support vector machine and xgboost) were used to predict identified dietary patterns based on sociodemographic and lifestyle factors.

**Results:**

After comparison, the optimal clustering method, K-means clustering, identified two distinct dietary patterns for both sexes, i.e. Traditional and Health-conscious patterns. The Traditional pattern was characterised by a higher energy intake and consumption of bread, potatoes, red and processed meat, coffee, fats and oils, and sugary drinks. Conversely, a higher intake of fruit, vegetables, tea, nuts, seeds, and breakfast cereals characterised the Health-conscious pattern. The classification models demonstrated moderate predictive accuracies (60–68%). According to the classifiers, the most important predictors for both sexes were education level, age, and BMI.

**Conclusion:**

Machine learning algorithms can be useful in identifying dietary patterns in population studies. We identified Health-conscious and Traditional patterns in a Dutch population, suggesting tailored public health interventions towards individuals adhering to a Traditional pattern. Future research should improve model validity and reproducibility to enhance its applicability in public health interventions and dietary guidelines.

**Supplementary Information:**

The online version contains supplementary material available at 10.1007/s00394-025-03817-4.

## Introduction

Dietary guidelines are developed with the aim of promoting a healthy diet and enhancing public health [1]. Dietary guidelines are formed primarily based on evidence from observational data studying the relationship between foods and health outcomes, including prospective cohort studies in addition to some randomised controlled trials [2]. The evidence behind dietary guidelines originates from the field of nutritional epidemiology, in which research explores the interplay between diet and health and disease in humans at the population level [3]. There are various perspectives from which the diet may be examined, including nutrient intake, foods, food groupings, and dietary patterns [3]. While most studies focus on the relationship between individual nutrients or foods and health outcomes, over the past two decades, there has been a shift towards studying dietary patterns, defined as measurements of typical food and nutrient combinations consumed by individuals and groups [4]. This shift is due to the recognition that individuals do not consume a single nutrient or food, but rather a meal consisting of a wide range of foods that include combinations of nutrients that may work in concert rather than single, isolated nutrients [4].

Traditional methods for identifying dietary patterns are a priori* (research-driven),* such as dietary index scores [5], a posteriori* (data-driven)*, such as principal component analysis (PCA) [6] and cluster analysis, and hybrid methods, such as reduced rank regression (RRR) [7], combining a priori and a posteriori [4, 8, 9]. However, the potential for synergistic effects among dietary components is not always considered in those methods [10]. Coding the interactions between dietary components into a statistical model is challenging using standard parametric approaches [10]. The failure to accurately capture heterogeneity in the population can result in formulating generalised dietary recommendations that do not capture diverse population needs, thus highlighting the need for a different approach.

Machine learning (ML) algorithms might aid in translating complex food patterns into quantifiable, explainable summaries [10]. K-means clustering has been applied to identify dietary patterns within specific populations [11–13]. Nevertheless, little information is provided about the rationale behind selecting K-means clustering and the optimal number of clusters [14]. In addition to the limited variations in clustering methods for dietary pattern analysis, dietary patterns are subject to change over time. Shifts in dietary patterns may be accelerated by global events, such as COVID-19. Given the worldwide impact of COVID-19, it is plausible that dietary patterns have changed. For instance, a systematic review by González-Monroy et al. [15] found that, due to COVID-19, adherence to healthy diets and the preference for nutritious foods, such as vegetables and fruits, declined. Recent research in the Netherlands further indicated that, although most adults did not change their dietary behaviour during the COVID-19 lockdown, certain socio-demographic groups, including individuals with overweight or obesity and those with a higher education level, were more likely to report eating less healthily compared to those with a healthy weight [16]. This finding underscores the importance of monitoring shifts in dietary patterns, particularly among vulnerable subgroups. However, all previously mentioned studies utilised dietary data collected before COVID-19. Additionally, the identification of dietary patterns using cluster analysis has not been conducted for the Dutch population.

Furthermore, to gain insights into which dietary patterns are related to which types of individuals, individuals could be classified based on personal characteristics into one of the derived dietary patterns. Classification is a supervised ML technique that attempts to predict the correct label for a given input dataset [17]. This approach facilitates the identification of the most important predictors of dietary patterns. For instance, Silva et al. [11] employed various ML classifiers to examine if these classifiers could predict the dietary patterns of the individuals based on sociodemographic and lifestyle factors.

Thus, we aimed to (1) apply and compare various clustering algorithms to identify dietary patterns in the Dutch general population using data from the latest Dutch National Food Consumption Survey (DNFCS); (2) classify individuals based on the personal features present and identify the important factors for the identified dietary patterns.

## Materials and methods

### Study population

The DNFCS is a representative survey of a representative Dutch population (children and adults) conducted approximately every two years. The DNFCS provides information on what, where, and when Dutch people eat and drink and is conducted by the National Institute for Public Health and the Environment (RIVM). Dietary and anthropometric information, socio-demographics, and lifestyle factors were collected in DNFCS [18]. We used the most recent survey, conducted between June 2019 and July 2021, for which 3570 individuals were recruited. We only included participants aged 18 years or older, as the energy intakes in children differ more between ages than in adults [19], resulting in a study population of 1747 individuals. The data from the 2019 DNFCS were available on request [20]. The dietary assessment and additional factors described below were all derived from the DNFCS. The data used in this report was derived from the DNFCS (2019–2021).

### Dietary assessment and food groups

The dietary assessment was conducted utilising two non-consecutive 24 h dietary recalls. The interview programme GloboDiet was employed to standardise the interviews and directly enter the answers into a computer [21]. The 24 h recalls were conducted via telephone or in-person interview, when necessary. The documented foods were classified using the GloboDiet classification of food groups, consisting of 18 main groups, 75 subgroups and 60 sub-subgroups. For the purpose of this study, we modified the food groups and ended with 29 food groups for the analysis (Supplementary Table S1). The quantity of food consumed by each participant per day, classified according to pre-defined food groups, was expressed in grams [22].

### Socio-demographic and lifestyle factors

The participants completed a questionnaire to provide information regarding their sociodemographic factors, lifestyle factors, and general dietary characteristics. The factors included in the analysis were sex, age, degree of urbanisation (extremely, hardly, or not urbanised), region (northern, eastern, southern, and western), migration background (Dutch, Western, Non-Western, and Unknown), household size, educational level, physical activity, screen time, smoking, alcohol consumption and BMI. The participants’ highest completed education level was classified into three categories: low (primary education, lower vocal education, and advanced elementary education), middle (intermediate vocational education and higher secondary education), and high (higher vocational education and university). Based on the number of days per week with at least adequate moderately intense exercise, physical activity was divided into three groups: inactive: < 1 day/week, semi-active: 1–5 days/week, and normally-active:   ≥5 days/week, considering 150 min of moderate-intensity activities weekly, such as commuting, leisure activities (walking, swimming), work, and household activities. Screen time was divided into three levels based on weekly hours: few (< 3.5 h), moderate (3.5–14 h), and many (> 14 h). Smoking status was categorised as smoker or non-smoker, alcohol consumption as user or non-user, and BMI was calculated by dividing weight by height squared (kg/m^2^). For comprehensive information on the survey methodology, please consult the detailed report provided by the RIVM [22].

### ML algorithms

A detailed parametrisation of the data cleaning and preprocessing can be found in Supplementary Methods S1.

#### Clustering algorithms

Four clustering algorithms (e.g., K-means, K-medoids, hierarchical, and density-based clustering) were employed to identify dietary patterns among males and females separately. These models were characterised and proposed as useful for dietary pattern analysis [10]. The following R packages were installed and executed: cluster, factoextra, dbscan, fpc, NbClust, and clValid. A detailed parametrisation of the clustering algorithms can be found in Supplementary Methods S2–S3.

The clustering algorithms were compared based on internal validation metrics, including the Silhouette Index (ranging from − 1 to 1, with values closer to 1 indicating better performance), the Davies-Bouldin (DB) Index (ranging from 0 to infinity, with lower values indicating better performance), the Dunn Index (ranging from 0 to infinity, with maximum values being optimal), and the Calinski-Harabasz (CH) Index (higher values are preferable, with no established cut-off value), as they could be calculated for all clustering methods [23–26]. In general, higher Silhouette, Dunn, and CH Index values and lower DB Index values indicate better clustering performance. No strict cut-off values were applied, and the relative performance of each method across all metrics was considered. Internal validation was used because the DNFCS data lacked labels and the "ground truth" for external validation [27]. In addition, cluster size balance, the cluster numbers, and the plausibility of the identified dietary patterns were compared. Based on all these measures, one optimal clustering algorithm was selected for both males and females. The internal validation metrics were calculated using the R functions, *silhouette*, *index.DB*, and *dunn*.

Furthermore, the results of the most effective algorithm were interpreted to determine which food groups were consumed more frequently by the various clusters, after which these clusters were labelled.

#### Classification algorithms

In this study, the dietary patterns identified by the optimal clustering algorithm were used to classify males and females in one of the patterns, meaning the clusters were the target variable. The factors included in the classification were age, education level, migration background, household size, BMI, smoking status, alcohol use, screen time, physical activity level, urbanisation level, and region. Most of these factors were categorical, with age and BMI being numerical variables.

The dataset was split into a training (70%) and a testing set (30%) [28]. Six different classifiers, namely Decision Tree (DT), Random Forest (RF), Naïve Bayes (NB), Support Vector Machine (SVM), K-nearest Neighbours (KNN), and Extreme Gradient Boosting package (XgBoost), were applied as classification methods [11, 17]. Tenfold cross-validation was employed throughout the training phase to ensure robustness and tune the model’s hyperparameters. A detailed parametrisation of the classification algorithms can be found in Supplementary Methods S4.

All classification models were assessed based on performance measures, including accuracy, specificity, sensibility, and negative and positive predictive value, by creating a confusion matrix using the *confusionMatrix* function. Feature importance was calculated using the *VarImp* function for SVM, KNN, NB, and RF, the *variable.importance* function for DT, and the *xgb.importance* function for XgBoost.

#### Statistical analysis

The categorical variables are reported as frequencies, while the continuous variables are reported as medians with interquartile ranges, as most continuous variables exhibited a non-normal distribution. Median food intakes per food group and participant characteristics were compared between dietary patterns using Median tests for continuous variables and Fisher’s Exact test for categorical variables. All analyses were conducted using R version 4.4.2.

## Results

Table 1 presents the characteristics of the study population. The median age of the participants was 57 years, and the median BMI value was 26.6 kg/m^2^. The majority of participants were of Dutch origin (86.5%) and had a high (38.3%) or middle (36.2%) education level. For males, a higher proportion of alcohol users was observed (78.3% vs. 67.0%) and a higher proportion of individuals with a high education level (42.0% vs. 34.6%).

**Table 1:** Characteristics of the study population, dutch national food consumption survey (DNFCS), 2019–2021

Variable	Total (N = 1733)	Male (N = 867)	Female (N = 866)
Age (years)^a^	57.0 [45.0, 68.0]	58.0 [44.0, 68.0]	57.0 [45.0, 67.0]
BMI (kg/m^2^)^a^	25.7 [23.3, 29.3]	26.0 [23.8, 28.7]	25.5 [22.6, 29.7]
Household size^a^	2.0 [2.0, 3.0]	2.0 [2.0, 3.0]	2.0 [1.0, 3.0]
*Education level*			
Low	441 (25.4%)	192 (22.1%)	249 (28.8%)
Middle	628 (36.2%)	311 (35.9%)	317 (36.6%)
High	664 (38.3%)	364 (42.0%)	300 (34.6%)
*Migration background*			
Dutch	1499 (86.5%)	745 (85.9%)	754 (87.1%)
Western	112 (6.5%)	60 (6.9%)	52 (6.0%)
Non-Western	108 (6.2%)	55 (6.3%)	53 (6.1%)
Unknown	14 (0.8%)	7 (0.8%)	7 (0.8%)
*Region of household*			
West	772 (44.5%)	377 (43.5%)	395 (45.6%)
North	172 (9.9%)	84 (9.7%)	88 (10.2%)
East	387 (22.3%)	201 (23.2%)	186 (21.5%)
South	402 (23.2%)	205 (23.6%)	197 (22.7%)
*Urbanisation level*			
Extremely	940 (54.2%)	466 (53.7%)	474 (54.7%)
Moderately	285 (16.4%)	143 (16.5%)	142 (16.4%)
Hardly	508 (29.3%)	258 (29.8%)	250 (28.9%)
*Alcohol use*			
Yes	1259 (72.6%)	679 (78.3%)	580 (67.0%)
No	474 (27.4%)	188 (21.7%)	286 (33.0%)
*Smoking*			
Yes	237 (13.7%)	117 (13.5%)	120 (13.9%)
No	1496 (86.3%)	750 (86.5%)	746 (86.1%)
*Physical activity*			
Inactive	55 (3.2%)	26 (3.0%)	29 (3.3%)
Semi-active	372 (21.5%)	194 (22.4%)	178 (20.6%)
Norm-active	1306 (75.4%)	647 (74.6%)	659 (76.1%)
*Screen time*			
Few (< 3.5 h/week)	19 (1.1%)	7 (0.8%)	12 (1.4%)
Moderate (3.5–14 h/week)	329 (19.0%)	164 (18.9%)	165 (19.1%)
Many (> 14 h/week)	1385 (79.9%)	696 (80.3%)	689 (79.6%)

BMI, body mass index. ^a^ Median and interquartile range

The evaluation metrics of the various clustering algorithms in identifying dietary patterns for males and females are presented in Table 2. Among males, K-medoids and Hierarchical Ward yielded three clusters, while K-means yielded two. The three clusters obtained for Hierarchical Ward were significantly imbalanced (one large cluster vs. two small clusters). In females, the optimal number of clusters was two for all clustering algorithms, and all cluster sizes were balanced. For both males and females, K-means attained the highest values for both the Silhouette score (0.045 and 0.054, respectively) and the CH Index (38.913 and 41.686, respectively), while for females, K-means also achieved the highest Dunn Index (0.116) (Table 2). The K-medoids clustering algorithm obtained the lowest DB Index for males (2.712) and females (2.631) (Table 2). Generally, higher Silhouette, Dunn, and CH Index values, and lower DB Index values are preferred. No strict cut-off values were applied; instead, the relative performance of each algorithm across all metrics was considered, along with the balance and interpretability of the clusters. Based on the overall combination of these measures, the K-means clustering algorithm was identified as the optimal choice for clustering based on this dataset.

**Table 2:** Clustering evaluation metrics by clustering algorithm and sex

	Male			Female		
Evaluation metric*	Kmeans	Kmedoids	Hierarchical ward	Kmeans	Kmedoids	Hierarchical ward
Number of clusters	2	3	3	2	2	2
Cluster sizes	477, 390	302, 193, 372	787, 37, 43	532, 334	393, 473	592, 274
Silhouette score	0.045	0.023	0.092	0.054	0.033	0.052
Dunn index	0.115	0.128	0.157	0.116	0.113	0.131
DB index	4.697	2.712	2.657	4.462	2.631	5.632
CH index	38.913	24.188	21.698	41.686	28.802	24.475

*For all metrics: higher Silhouette, Dunn, and CH index values and lower DB index values indicate better clustering performance. DB index, davies-bouldin index; CH index, calinski-harabasz

The dietary patterns identified by K-means clustering for males and females are shown in Table 3. Compared with the Traditional dietary pattern, the Health-conscious pattern was characterised by substantially greater consumption of both fruit (87% higher in males and 48% in females) and vegetables (increased by 39% in males and 65% in females) (Table 3). Conversely, the Traditional pattern showed substantially higher intakes of sugary drinks and processed meat in both sexes. For example, sugary drink consumption was more than double among males (325 vs. 124 mL/day) within the Traditional group. Processed meat intake was also considerably higher in the Traditional group (75 vs. 33 g/day for males; 56 vs. 1 g/day for females) (Table 3). Additional sex-specific differences included higher intakes of baked and frozen sweets (+ 43%) and sweetened dairy (30 vs. 0 g/day) among females following the Traditional pattern, whereas males with a Health-conscious pattern had a greater consumption of unsweetened dairy (+ 35%) (Table 3). A comparison of males’ and females’ dietary intakes revealed that males consumed higher quantities of several food items, including sugary drinks, meat (both red and processed), potatoes, savoury sauces, fats and oils, coffee, and bread, resulting in a higher median energy intake.

**Table 3:** Food consumption by dietary pattern and sex, Dutch National Food Consumption Survey (DNFCS), 2019–2021

	Male		Female	
Food group (g or mL day^−1^)	Traditional (N = 477)	Health-conscious (N = 390)	Traditional (N = 532)	Health-conscious (N = 334)
Energy^a^	2284.95** [1974.07, 2733.22]	2096.15 [1790.24, 2512.87]	1799.60** [1520.68, 2091.88]	1690.18 [1375.52, 1998.34]
Alcoholic beverages	0.00 [0.00, 352.50]	0.00 [0.00, 271.56]	0.00 [0.00, 130.65]	0.00 [0.00, 125.00]
Baked and frozen sweets	60.00 [22.50, 110.00]	53.46 [5.25, 104.14]	57.00* [24.00, 103.62]	40.25 [1.75, 82.00]
Bread	151.50** [108.50, 210.00]	123.75 [82.62, 171.79]	106.75* [75.00, 147.56]	90.25 [52.82, 130.00]
Cheese	37.83 [19.50, 64.56]	37.22 [20.00, 62.00]	36.00 [22.00, 58.25]	31.00 [15.83, 52.49]
Coffee	550.00* [316.67, 800.00]	445.00 [225.42, 659.63]	443.33** [168.87, 663.95]	280.00 [4.12, 500.00]
Eggs	0.00 [0.00, 45.00]	0.00 [0.00, 50.00]	0.00 [0.00, 47.50]	0.00 [0.00, 50.00]
Fats and oils	27.55** [17.44, 39.38]	22.23 [13.03, 31.66]	18.00* [11.27, 28.70]	14.37 [8.83, 22.45]
Fish	0.00** [0.00, 0.00]	0.00 [0.00, 68.91]	0.00* [0.00, 12.50]	0.00 [0.00, 69.99]
Fruits	114.00** [0.00, 177.38]	213.34 [120.00, 299.23]	133.98** [62.95, 208.26]	197.80 [130.00, 286.23]
Potatoes	143.00** [64.67, 218.00]	62.00 [0.00, 141.38]	104.75** [34.20, 143.50]	12.44 [0.00, 105.00]
Processed meat	74.69** [37.80, 116.00]	32.53 [11.00, 74.36]	56.12** [23.00, 88.53]	0.69 [0.00, 30.00]
Red meat	46.45** [0.00, 111.00]	0.00 [0.00, 67.12]	20.33** [0.00, 78.00]	0.00 [0.00, 15.62]
Savoury sauces	45.95** [18.62, 75.25]	17.23 [0.00, 42.00]	26.97** [10.06, 53.16]	11.64 [0.00, 30.00]
Unsweetened dairy	200.80** [20.00, 346.77]	271.61 [114.63, 438.01]	206.95 [53.29, 343.41]	197.47 [59.48, 324.00]
Vegetables	142.15** [96.66, 194.62]	197.86 [136.05, 268.30]	134.89** [89.73, 187.93]	222.61 [146.53, 312.03]
Soups	0.00 [0.00, 11.00]	0.00 [0.00, 11.64]	0.00* [0.00, 1.05]	0.00 [0.00, 10.96]
Sweetened dairy	12.00** [0.00, 206.00]	0.00 [0.00, 123.28]	29.77** [0.00, 206.00]	0.00 [0.00, 2.38]
Tea	0.00** [0.00, 325.00]	400.00 [0.00, 824.17]	365.42** [0.00, 680.21]	645.00 [275.00, 1104.69]
Meat and dairy substitutes	0.00** [0.00, 0.00]	0.00 [0.00, 0.00]	0.00** [0.00, 0.00]	0.00 [0.00, 84.16]
Nuts and seeds	0.00** [0.00, 30.00]	19.66 [0.00, 39.00]	0.00** [0.00, 22.80]	17.58 [0.00, 36.25]
Pasta, rice, other grain	0.00 [0.00, 155.90]	56.25 [0.00, 173.43]	0.00** [0.00, 110.00]	60.00 [0.00, 139.95]
Savoury bread spreads	0.00 [0.00, 0.00]	0.00 [0.00, 0.00]	0.00 [0.00, 0.00]	0.00 [0.00, 2.78]
Spices and herbs	0.00* [0.00, 4.80]	0.00 [0.00, 9.49]	0.00* [0.00, 3.60]	0.00 [0.00, 8.04]
Breakfast cereals	0.00** [0.00, 0.00]	11.48 [0.00, 40.00]	0.00** [0.00, 2.93]	9.84 [0.00, 34.02]
White meat	0.00 [0.00, 50.86]	0.00 [0.00, 59.28]	0.00* [0.00, 20.38]	0.00 [0.00, 56.25]
Savoury snacks	0.00** [0.00, 60.00]	0.00 [0.00, 19.00]	0.00 [0.00, 36.14]	0.00 [0.00, 25.00]
Legumes	0.00* [0.00, 0.00]	0.00 [0.00, 0.00]	0.00** [0.00, 0.00]	0.00 [0.00, 0.00]
Confectionery	27.00 [7.65, 53.61]	21.21 [4.76, 45.73]	22.18 [7.00, 44.77]	18.51 [3.77, 35.98]
Sugary drinks	325.00** [0.00, 656.00]	123.60 [0.00, 307.69]	179.39** [0.00, 462.19]	6.25 [0.00, 212.74]

* (*p* < 0.05) and ** (*p* < 0.001) represent *p* values derived from Median tests between the traditional and health-conscious patterns. ^a^ kcal day^−1^

Table 4 presents the characteristics of the study population by pattern and sex. For both sexes, the Traditional pattern was characterised by a higher mean BMI (kg/m^2^), a higher proportion of individuals with a low education level and a higher proportion of smokers. For males, the Traditional pattern also had a higher proportion of individuals from the north of the Netherlands. Among females, the Traditional pattern was distinguished by a higher proportion of inactive and semi-active individuals, a higher proportion of individuals of Dutch origin, and a higher proportion of individuals with a screen time higher than 14 h. Conversely, the health-conscious pattern was distinguished by a higher proportion of individuals with a high education level, a lower mean BMI, and a higher proportion of non-smokers, irrespective of sex.

**Table 4:** Characteristics of the study population by dietary pattern and sex

	Male		Female	
Variable	Traditional (N = 477)	Health-conscious (N = 390)	Traditional (N = 532)	Health-conscious (N = 334)
Age (years)^a^	58.0 [44.0, 67.0]	58.5 [45.0, 70.0]	57.0 [45.0, 67.0]	57.0 [43.25, 66.75]
BMI (kg/m^2^)^a^	26.3* [24.3, 29.7]	25.4 [23.325, 28.1]	26.1** [23.2, 30.4]	24.5 [21.9, 28.1]
Household size^a^	2.0 [2.0, 3.0]	2.0 [2.0, 3.0]	2.0 [2.0, 3.0]	2.0 [1.0, 3.0]
*Education level*				
Low	131 (27.5%)**	61 (15.6%)	191 (35.9%)**	58 (17.4%)
Middle	185 (38.8%)	126 (32.3%)	210 (39.5%)	107 (32.0%)
High	161 (33.8%)	203 (52.1%)	131 (24.6%)	169 (50.6%)
*Migration background*				
Dutch	420 (88.1%)	325 (83.3%)	481 (90.4%)*	273 (81.7%)
Western	32 (6.7%)	28 (7.2%)	25 (4.7%)	27 (8.1%)
Non-Western	23 (4.8%)	32 (8.2%)	23 (4.3%)	30 (9.0%)
Unknown	2 (0.4%)	5 (1.3%)	3 (0.6%)	4 (1.2%)
*Urbanisation level*				
Extremely	244 (51.2%)	222 (56.9%)	281 (52.8%)	193 (57.8%)
Moderately	81 (17.0%)	62 (15.9%)	95 (17.9%)	47 (14.1%)
Hardly	152 (31.9%)	106 (27.2%)	156 (29.3%)	94 (28.1%)
*Region of household*				
West	189 (39.6%)*	188 (48.2%)	235 (44.2%)	160 (47.9%)
North	59 (12.4%)	25 (6.4%)	54 (10.2%)	34 (10.2%)
East	108 (22.6%)	93 (23.8%)	122 (22.9%)	64 (19.2%)
South	121 (25.4%)	84 (21.5%)	121 (22.7%)	76 (22.8%)
*Alcohol use*				
Yes	367 (76.9%)	312 (80.0%)	356 (66.9%)	224 (67.1%)
No	110 (23.1%)	78 (20.0%)	176 (33.1%)	110 (32.9%)
*Smoking*				
Yes	80 (16.8%)*	37 (9.5%)	92 (17.3%)**	28 (8.4%)
No	397 (83.2%)	353 (90.5%)	440 (82.7%)	306 (91.6%)
*Physical activity*				
Inactive	20 (4.2%)	6 (1.5%)	23 (4.3%)*	6 (1.8%)
Semi-active	107 (22.4%)	87 (22.3%)	123 (23.1%)	55 (16.5%)
Norm-active	350 (73.4%)	297 (76.2%)	386 (72.6%)	273 (81.7%)
*Screen time*				
Few (< 3.5 h/week)	4 (0.8%)	3 (0.8%)	10 (1.9%)*	2 (0.6%)
Moderate (3.5–14 h/week)	77 (16.1%)	87 (22.3%)	85 (16.0%)	80 (24.0%)
Many (> 14 h/week)	396 (83.0%)	300 (76.9%)	437 (82.1%)	252 (75.4%)

* (*p* < 0.05) and ** (*p* < 0.001) represent *p* values derived from Median and Fisher’s Exact tests between the traditional and health-conscious patterns. BMI, body mass index. ^a^ Median and interquartile range

The performance of the six classifier models–DT, RF, NB, KNN, SVM, and XgBoost–are presented in Table 5. For males, NB and SVM achieved the highest accuracy (0.62, 95% CI 0.56–0.68), followed by RF (0.61, 95% CI 0.55–0.67). RF achieved the highest sensitivity (0.83) and negative predictive value (0.62). However, it is important to note that the specificity levels were relatively low across all models, with the highest value being 0.59 for DT. In the female group, SVM attained the highest accuracy (0.68, 95% CI 0.62–0.74), followed by DT with an accuracy of 0.67 (95% CI 0.61–0.73) and KNN, and RF with an accuracy of 0.66. The SVM also achieved the highest negative predictive value (0.62). The highest sensitivity was observed for KNN (0.85), while the highest positive predictive value was achieved by DT (0.72). Notably, the specificity levels were comparatively low across all models, with the highest recorded being 0.52 for DT. Overall, higher accuracies were obtained for the female dataset.

**Table 5:** Comparison of six classification models by different evaluation metrics in males and females

Metric	DT	RF	NB	KNN	SVM	XgBoost
*Males*						
Accuracy	0.60	0.61	0.62	0.59	0.62	0.60
95% CI	(0.54, 0.66)	(0.55, 0.67)	(0.56, 0.68)	(0.53, 0.65)	(0.56, 0.68)	(0.54, 0.66)
Sensitivity	0.60	0.83	0.77	0.73	0.74	0.78
Specificity	0.59	0.35	0.44	0.43	0.47	0.38
Pos. predictive value	0.79	0.61	0.63	0.61	0.63	0.61
Neg. predictive value	0.37	0.62	0.61	0.56	0.60	0.59
*Females*						
Accuracy	0.67	0.66	0.64	0.66	0.68	0.63
95% CI	(0.61, 0.73)	(0.60, 0.72)	(0.57, 0.69)	(0.60, 0.72)	(0.62, 0.74)	(0.57, 0.69)
Sensitivity	0.76	0.79	0.78	0.85	0.83	0.71
Specificity	0.52	0.45	0.42	0.35	0.44	0.51
Pos. predictive value	0.72	0.70	0.67	0.68	0.70	0.70
Neg. predictive value	0.58	0.58	0.55	0.59	0.62	0.53

95% CI, 95% confidence interval

For the models, NB, KNN, and SVM, education level and BMI were identified as the most important features for both males and females (Supplementary Table S2–S3). However, the magnitude of these importances exhibited a narrow range, with values ranging from 0.51 to 0.60 for the male dataset and from 0.51 to 0.66 for the female dataset. The DT, RF, and XgBoost models identified BMI and age as the most important features in the male dataset. Conversely, for females, the models demonstrate that for DT, education level was the most important feature, while for RF and XgBoost, BMI and age were the most important features. Overall, individual factors and mainly socio-demographic factors were the most important factors.

## Discussion

The present study aimed to compare ML algorithms in identifying dietary patterns and find important characteristics classifying the identified dietary patterns within a Dutch general population. Comparing various clustering algorithms identified two dietary patterns: the Traditional and Health-conscious patterns for males and females. For both sexes, the Health-conscious pattern was characterised by a high consumption of fruit, vegetables, nuts and seeds, and breakfast cereals. Furthermore, males with a health-conscious pattern had a high intake of unsweetened dairy, while females with a Health-conscious pattern had a high consumption of pasta, rice and other grains. Conversely, the Traditional pattern was characterised by high consumption of bread, coffee, fats and oils, sugary drinks for both sexes, red and processed meat for males, and potatoes for females. When utilising six distinct classification methods with moderate accuracy (60–68%), it was identified that individuals with higher education levels, lower BMI, no smoking, and lower age are more likely to have a Health-conscious dietary pattern in males. Individuals with higher education levels, lower BMI, lower age, and smaller household size are more likely to have a Health-conscious dietary pattern in females.

This study compared four clustering algorithms and determined that K-means clustering was the most appropriate clustering algorithm for identifying two distinct dietary patterns for both sexes. Our results correspond to previous studies comparing various clustering algorithms in dietary intake data that K-means clustering was the optimal approach compared to other clustering algorithms [7, 14]. To our knowledge, no studies used K-medoids or density-based clustering for nutritional data. It was hypothesised that K-medoids would be more resistant to noise [29] and that density-based clustering would handle clusters of any shape [17], thus making it useful for complex nutritional data. However, in our study, K-medoids’ efficacy in effectively handling noise was not evidenced by its performance compared to the K-means clustering, likely due to the already effective management of outliers during the data preparation stage.

According to the K-means clustering algorithm, the identified dietary patterns were the Traditional pattern and the Health-conscious pattern (fruit, vegetables, nuts and seeds) for both sexes. The Traditional and Health-conscious patterns identified in the present study correspond to those found more frequently in several European countries [30–34]. For example, Waijers et al. [35] identified three patterns in Dutch women between 60 and 69 years using PCA. The identified patterns were a Mediterranean-like pattern (pasta, rice), a Traditional Dutch pattern (potatoes, meat), and a healthy Traditional Dutch pattern (fruit, vegetables, and dairy products). This finding aligns, in part, with the dietary patterns observed among female participants in this study, where the health-conscious pattern is a combination of the Mediterranean-like and healthy Traditional Dutch pattern. Another article by Heerschop et al. [36] investigated the dietary patterns of the Dutch general population using the DNFCS of 2016–2019 with a sample size of 2078. The researchers employed reduced rank regression to identify three distinct patterns. The first pattern was characterised by high consumption of fruit and vegetables, the second by low meat consumption, and the third by low consumption of fruit juices and high dairy consumption [36]. It was observed that these dietary patterns corresponded to the health-conscious pattern for both sexes. The absence of any pattern corresponding with the identified Traditional pattern suggests that during the data collection period (COVID-19), the Dutch population may have adopted a slightly less healthy diet. This aligns with international findings, such as those by González-Monroy et al. [15], who reported a decline in adherence to healthy diets during COVID-19. In the Netherlands, it is also observed that certain groups, including those with overweight/obesity and higher education, reported less healthy eating behaviours during lockdown [16].

Differences in sociodemographic and lifestyle characteristics were observed across the identified dietary patterns. As observed in previous studies, males and females within the healthy pattern demonstrated a higher level of education, a lower BMI, and a lower proportion of smokers [14, 30–34, 37, 38]. As exhibited in several articles, a higher proportion of individuals engaging in higher physical activity levels was reported for the healthy pattern [14, 30, 34, 37, 38]. However, these results were exclusively observed among females in the present study. Conversely, a higher proportion of males in the Traditional pattern was observed to originate from the northern regions of the Netherlands. This result could be attributed to the lower socioeconomic status and income levels in the north of the Netherlands [39, 40].

The six classifiers demonstrated the capacity to classify individuals into one of the identified dietary patterns based on sociodemographic and lifestyle factors with moderate accuracies (60–68%). The lack of research utilising ML techniques to predict dietary patterns using demographic data limits the ability to compare our results with those of prior research. Silva et al. [11] investigated the same six classifiers to predict dietary patterns in 12,677 Brazilian adults aged 35–74 years using sociodemographic and clinical features. The accuracy of each algorithm’s ability to classify individuals ranged from 69 to 72%. The accuracies observed in the present study (60–68%) were lower than those reported in the study mentioned above. However, this discrepancy could be attributed to this study’s significantly higher sample size (n = 12,677 vs. n = 866 or 867), cultural considerations, or other methodological issues. Overall, these predictive accuracies can be considered moderate. As the use of classification models in this context is still relatively novel, there are no established standards for acceptable performance. Therefore, all the model performance metrics should be interpreted as exploratory and potentially useful as reference values for future research. Additionally, some models, such as RF for males, showed high sensitivity but low specificity for identifying the Health-conscious pattern. This imbalance may be partly attributed to the modest class imbalance present in the dataset, which was not corrected through resampling or algorithmic class weighting [41]. Additionally, the dominant influence of features, such as education level and BMI may have disproportionately driven the classification decisions, potentially overshadowing other discriminative variables. For example, lower BMI and higher education level were more strongly associated with the Health-conscious cluster and may have led to biased predictions toward this class, contributing to the observed imbalance. We chose not to apply class balancing techniques in this initial analysis to preserve the real-world distribution of the data and to avoid introducing synthetic variability. Nonetheless, we recognise that these methodological choices may affect the model’s ability to generalise, and they should be considered when interpreting the results.

The classifications were primarily based on education level, age, and BMI for both sexes. Education, BMI, and age are well-known factors associated with dietary patterns [30, 33, 34, 42]. Our results partially correspond to Silva et al. [11] that per capita income, sex, education level, age, and physical activity emerged as the most important features for classifying Brazilian adults into their identified dietary patterns. Moreover, household size and screen time were also important factors identified for females, while smoking had emerged for males. One potential explanation for this observation is that a larger household size may correspond with a reduced financial budget per person, which may result in a preference for less healthy foods, often perceived as less expensive. The importance of smoking for males may be attributed to its relationship with a less healthy overall lifestyle [43]. In addition, Fransen et al. [42] investigated the association between education level, smoking, and dietary patterns in a Dutch adult population. The study found that current smokers with a low education level exhibited a less healthy dietary pattern compared to non-smokers. We also noted a higher proportion of smokers with a low education level for a Traditional pattern compared to a Health-conscious one. These findings suggest that although they do not demonstrate an association, BMI, educational level, age, household size, and smoking are important factors.

Our findings have several methodological and policy implications. In the context of ML algorithms, K-means clustering is recommended for dietary pattern analysis, yet its application requires methodological justification to ensure objectivity and reproducibility. Further research on the clustering algorithms is necessary to determine the consistency and validity of K-means clustering and to explore alternative methods, such as Gaussian Mixture Models. The overall moderate accuracy of the classification models (60–68%) suggests that multiple models are valuable for this research domain. The choice of classifier depends on the balance between explainability, simplicity, and data type. Further research on the classification models in this domain should examine whether sociodemographic and lifestyle factors are consistently associated with dietary patterns. Moreover, larger sample sizes could enhance the clustering and classification outcomes. Additionally, future studies should use longitudinal data to assess the repeatability and stability of the identified dietary patterns over time. Besides, this data could also be used to further elucidate causal relationships between sociodemographic/lifestyle factors and dietary pattern adherence. Additionally, the analyses on dietary patterns suggest that the Traditional pattern includes food groups associated with an elevated risk of adverse health outcomes, making it particularly relevant for public health interventions aimed at reducing the burden of chronic diseases, such as obesity and diabetes. Individuals adhering to a Traditional pattern may thus have an increased risk of developing certain chronic diseases. Public health interventions should target individuals with a Traditional pattern, encouraging them, for example, to reduce sugary drinks intake and increase vegetable consumption. As the Traditional pattern is more prevalent in people with lower education levels, higher BMI, and older age, these demographic groups should be considered in the interventions. However, these findings should be interpreted cautiously, as the classification models used in this study have methodological limitations, and the results are indicative rather than definitive.

The present study has several strengths. Firstly, it compared multiple clustering algorithms on the same dataset. Employing various clustering algorithms provides insights into the available methods for dietary pattern analysis. Secondly, the study sought to provide a comprehensive and objective workflow, which is particularly noteworthy given the subjective nature of cluster analysis. This systematic approach can be employed to explore dietary patterns in other populations. Thirdly, we identified dietary patterns separately for males and females, which gave insights into potential gender-specific differences in dietary patterns and important sociodemographic factors. Fourthly, we used dietary intake data from 2019 to 2021, which yielded insights into the shifts in dietary patterns that occurred before and during the pandemic. Finally, the study utilised six distinct classifiers to predict dietary patterns, providing a comprehensive overview of the available classifiers and their efficacy on this particular data set.

This study also has several limitations. Firstly, while clustering and classification methods can identify patterns and important sociodemographic and lifestyle factors, they do not provide insights into the biological mechanism by which disease and dietary intake are associated. Furthermore, due to the cross-sectional design, only associations can be examined, and causality cannot be inferred from the relationships observed between individual characteristics and dietary patterns. Secondly, the data from the DNFCS was collected using 24 h recall, which is considered to be a validated measure, nevertheless, it can introduce recall bias. Thirdly, a larger sample size and a more balanced age range could have yielded different outcomes for the density-based clustering algorithm. The dietary intake of the individuals was too similar to result in distinct regions of high density. Fourthly, the exclusion of younger participants limits the generalizability of our findings to children and adolescents. Future studies should include a broader age range to improve the applicability of results to the entire population. Lastly, no external validation was possible due to the absence of labels or other independent datasets to verify the findings. As a result, the reproducibility of our findings is limited, and future research is needed to confirm these results in other populations and settings.

## Conclusion

In conclusion, we demonstrated that K-means clustering was the optimal algorithm for this comparative and exploratory study, identifying two distinct dietary patterns among males and females in the Dutch population: a Health-conscious and a Traditional pattern. The classification models used to predict dietary patterns demonstrated moderate accuracy (60–68%), with key predictors including age, BMI, and education level for both sexes, smoking for males, and household size for females.

## Supplementary Information

Below is the link to the electronic supplementary material.

Supplementary Material 1
