# Supplementary material for: Identifying and predicting dietary patterns in the Dutch population using machine learning
Source: Eur J Nutr. 2025 Oct 23;64(8):305. doi: 10.1007/s00394-025-03817-4 (PMC12549413; doi:10.1007/s00394-025-03817-4)
Supplement: Supplementary file 1 — Supplementary Material 1 [file 394_2025_3817_MOESM1_ESM.docx]

**Supplementary Materials**

**Journal:** European Journal of Nutrition

**Title:** Identifying and Predicting Dietary Patterns in the Dutch Population Using Machine Learning

**Authors:** Marlijn L. van Houwelingen^1^, Yinjie Zhu^1,2^

^1^ Consumption and Healthy Lifestyles chair group, Wageningen University & Research, Hollandseweg 1, 6706 KN, Wageningen, the Netherlands;

^2^ National Institute for Public Health and the Environment, Antonie van Leeuwenhoeklaan 9, 3721 MA, Bilthoven, the Netherlands;

**Corresponding author:** Yinjie Zhu; email: [yinjie.zhu@wur.nl](mailto:yinjie.zhu@wur.nl)

**Contents:**

**Supplementary Table S1** Customised food groups based on GloboDiet and NEVO classification and domain knowledge.

**Supplementary Table S2** Feature importance per classifier for males

**Supplementary Table S3** Feature importance per classifier for females

**Supplementary Methods S1** Extra explanation of data cleaning and preprocessing

**Supplementary Methods S2** Extra explanation of unsupervised machine learning

**Supplementary Methods S3** Extra explanation of clustering algorithms

**Supplementary Methods S4** Extra explanation of classification algorithms

# **Table S1** Customised food groups based on GloboDiet and NEVO classification [1] and domain knowledge.

| Food groups |
| --- |
| Potatoes |
| Vegetables |
| Legumes |
| Fruits |
| Nuts & seeds |
| Sweetened dairy |
| Unsweetened dairy |
| Cheese |
| Bread ( + Dough & pastry) |
| Pasta, rice, other grain |
| Breakfast cereals |
| Red meat |
| White meat |
| Processed meat |
| Meat & dairy substitutes |
| Fish |
| Eggs |
| Fats & oils |
| Confectionery (confectionary, chocolate, sugars & sweet spreads) |
| Baked & Frozen sweets |
| Sugary drinks (Soft drinks, fruit & vegetable juices) |
| Coffee |
| Tea |
| Alcoholic beverages |
| Savoury sauces |
| Spices & herbs |
| Soups |
| Savoury snacks |
| Savoury bread spreads |

# **Table S2** Feature importance per classifier for males

| **Order** | **DT^a^** | | **RF^b^** | | **NB^c^** | | **KNN^c^** | | **SVM^c^** | | **XgBoost^d^** | |
| --- | --- | --- | --- | --- | --- | --- | --- | --- | --- | --- | --- | --- |
| **1** | BMI | 10.23 | BMI | 21.61 | Education level | 0.60 | Education level | 0.60 | Education level | 0.60 | BMI | 0.27 |
| **2** | Age | 10.05 | Age | 17.34 | BMI | 0.60 | BMI | 0.60 | BMI | 0.60 | Age | 0.26 |
| **3** | Migration background | 4.97 | Education level | 8.84 | Smoking status | 0.54 | Smoking status | 0.54 | Smoking status | 0.54 | Education level | 0.12 |
| **4** | Smoking status | 4.81 | Household size | 7.96 | Region | 0.53 | Region | 0.53 | Region | 0.53 | Region | 0.06 |
| **5** | Screen time | 0.23 | Region | 7.52 | Screen time | 0.52 | Screen time | 0.52 | Screen time | 0.52 | Smoking status | 0.06 |
| **6** | Physical activity level | 0.16 | Migration background | 5.81 | Household size | 0.52 | Household size | 0.52 | Household size | 0.52 | Household size | 0.05 |
| **7** | Household size | 0.08 | Urbanisation level | 5.30 | Physical activity level | 0.52 | Physical activity level | 0.52 | Physical activity level | 0.52 | Urbanisation level | 0.04 |
| **8** | Alcohol use | 0.00 | Screen time | 4.78 | Migration background | 0.52 | Migration background | 0.52 | Migration background | 0.52 | Physical activity level | 0.04 |
| **9** | Education level | 0.00 | Physical activity level | 4.75 | Urbanisation level | 0.52 | Urbanisation level | 0.52 | Urbanisation level | 0.52 | Migration background | 0.04 |
| **10** | Urbanisation level | 0.00 | Smoking status | 3.77 | Age | 0.51 | Age | 0.51 | Age | 0.51 | Alcohol use | 0.03 |
| **11** | Region | 0.00 | Alcohol use | 2.74 | Alcohol use | 0.51 | Alcohol use | 0.51 | Alcohol use | 0.51 | Screen time | 0.02 |

^a^ Feature importance was calculated based on the total of the split measures' goodness for every split for which it served as the main variable.

^b^ Feature importance was extracted based on the mean decrease in Gini impurity.

^c^ Feature importance was extracted based on the area under the curve (ROC).

^d^ Feature importance was extracted based on the Gain (increase in accuracy a feature provides to the branches it is on).

# **Table S3** Feature importance per classifier for females

| **Order** | **DT^a^** | | **RF^b^** | | **NB^c^** | | **KNN^c^** | | **SVM^c^** | | | **XgBoost^d^** | |
| --- | --- | --- | --- | --- | --- | --- | --- | --- | --- | --- | --- | --- | --- |
| **1** | Education level | 18.79 | BMI | 38.72 | Education level | 0.66 | Education level | 0.66 | Education level | 0.66 |  | BMI | 0.28 |
| **2** | Screen time | 1.16 | Age | 35.30 | BMI | 0.59 | BMI | 0.59 | BMI | 0.59 |  | Age | 0.24 |
| **3** | BMI | 0.54 | Education level | 21.96 | Household size | 0.56 | Household size | 0.56 | Household size | 0.56 |  | Education level | 0.11 |
| **4** | Smoking status | 0.00 | Household size | 19.44 | Physical activity level | 0.56 | Physical activity level | 0.56 | Physical activity level | 0.56 |  | Household size | 0.08 |
| **5** | Age | 0.00 | Region | 14.30 | Migration background | 0.54 | Migration background | 0.54 | Migration background | 0.54 |  | Region | 0.08 |
| **6** | Physical activity level | 0.00 | Urbanisation level | 10.60 | Smoking status | 0.54 | Smoking status | 0.54 | Smoking status | 0.54 |  | Urbanisation level | 0.06 |
| **7** | Household size | 0.00 | Migration background | 8.75 | Screen time | 0.53 | Screen time | 0.53 | Screen time | 0.53 |  | Migration background | 0.04 |
| **8** | Alcohol use | 0.00 | Physical activity level | 8.57 | Age | 0.53 | Age | 0.53 | Age | 0.53 |  | Physical activity level | 0.03 |
| **9** | Migration background | 0.00 | Screen time | 6.88 | Region | 0.53 | Region | 0.53 | Region | 0.53 |  | Smoking status | 0.03 |
| **10** | Urbanisation level | 0.00 | Smoking status | 6.50 | Alcohol use | 0.51 | Alcohol use | 0.51 | Alcohol use | 0.51 |  | Alcohol use | 0.02 |
| **11** | Region | 0.00 | Alcohol use | 5.03 | Urbanisation level | 0.51 | Urbanisation level | 0.51 | Urbanisation level | 0.51 |  | Screen time | 0.02 |

^a^ Feature importance was calculated based on the total of the split measures' goodness for every split for which it served as the main variable.

^b^ Feature importance was extracted based on the mean decrease in Gini impurity.

^c^ Feature importance was extracted based on the area under the curve (ROC).

^d^ Feature importance was extracted based on the Gain (increase in accuracy a feature provides to the branches it is on).

# **Methods S1** Extra explanation of data cleaning and preprocessing

The participant dataset included age, sex, BMI, education level, migration background, household size, urbanisation information, dietary habits, and lifestyle factors (e.g., smoking, physical activity, screen time and alcohol consumption). The relevant columns contained no missing values and required no imputation or deletion. Only relevant columns (individual identifiers, food products, food groups, and quantity consumed) were retained in the food consumption dataset, while unnecessary columns were removed. The datasets were merged based on participant ID, and participants younger than 18 were excluded.

The food groups were initially examined to determine whether the products were correctly classified. Subsequently, the food groups underwent customisation and merging based on the GloboDiet food groups, the Dutch Food Composition Database (NEVO), and domain knowledge. The misclassified products were then placed in the right customised food groups, resulting in 29 food groups (Appendix A).

The quantity consumed was aggregated by participant and food group, with values averaged in grams per day to prepare the data for machine learning. Individuals whose energy consumption was deemed implausible, falling below 500 kcal or exceeding 4,000 kcal per day [2], reduced the sample size by n = 13, resulting in a total sample size of n = 1,733. Non-consumption of a food group was stated as zero to ensure the completeness of the data. The data was structured as one row per participant and one column for every food group.

Before applying clustering algorithms, the data was transformed into z-scores to standardise variables with different units and ranges. Euclidean distance was used as the similarity measure, appropriate for continuous numerical data.

# **Methods S2** Extra explanation of unsupervised machine learning

ML is the process by which computers acquire specific skills without explicit instruction. One category of ML is unsupervised learning, which uses unlabelled data to identify hidden patterns within the dataset. Unsupervised ML uses similarities to identify common characteristics [21]. Clustering is one of the most common unsupervised ML techniques, with the most used clustering algorithms being partitioning methods (e.g., k-means and k-medoids), density-based, and hierarchical clustering [12]. Partitioning methods, e.g., K-means and K-medoids, divide the data into a predetermined number of spherical clusters according to the characteristics and commonalities in the data and try to minimise the within-cluster variance [12] (**Figure 1A**). The difference between the K-means and K-medoids algorithm is that K-medoids indicates a cluster centre by using a real point inside the cluster compared to the mean point, which makes K-medoids stronger against noises and anomalies [22]. In density-based clustering, the clusters consist of groups of high-density points, separated by regions of low-density points, typically seen as noise or outliers [23]. The most widely used density-based algorithm is the density-based spatial clustering of applications with noise (DBSCAN), which is not dependent on a predefined number of clusters and can locate clusters with any shape [12] (**Figure 1B**). Lastly, hierarchical clustering groups objects in clusters according to their similarity in an agglomerative (bottom-up) or divisive (top-down) way. Agglomerative clustering is the most frequently used method, initiated with N clusters for N samples. The clusters with the highest degree of similarity will be merged until the number of clusters is condensed to one, resulting in a dendrogram [24] (**Figure 1C**). Concerning nutritional epidemiology, unsupervised ML could identify clusters of individuals with analogous dietary patterns [25].


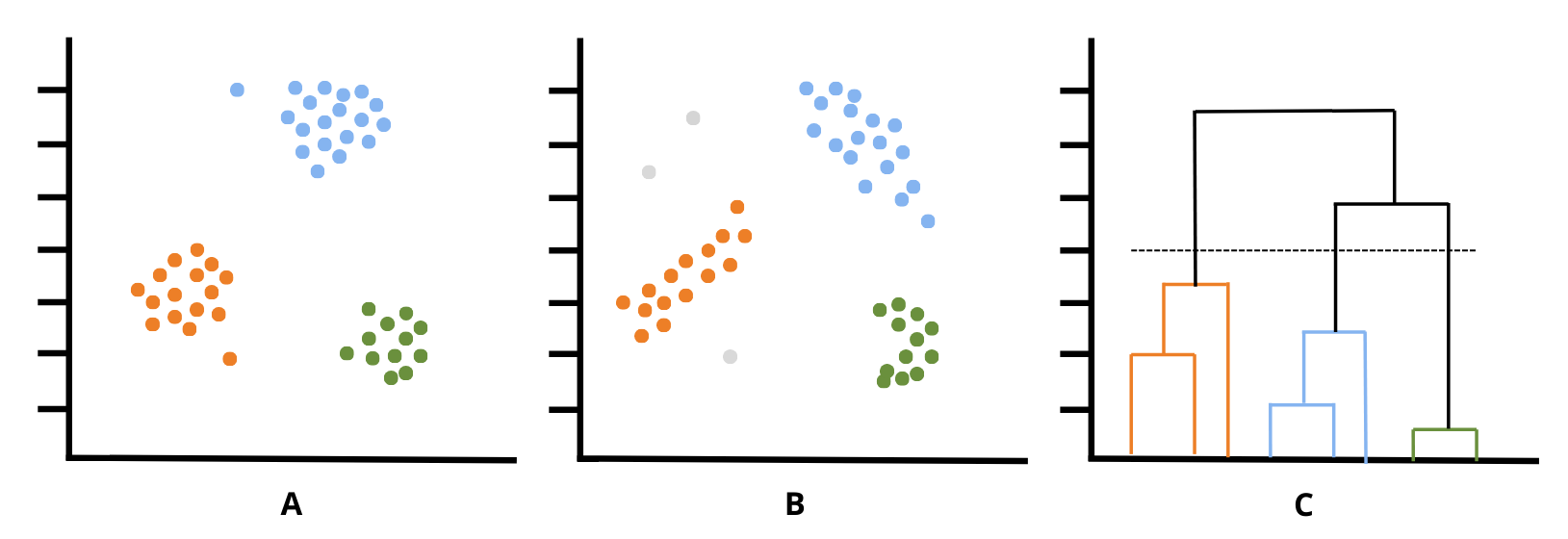


**Figure 1**: Example outcomes of different clustering methods with three clusters. A: partitioning methods (spherical clusters), B: density-based clustering (different shapes and grey dots are considered noise), and C: hierarchical clustering (dendrogram cut at three clusters).

# **Methods S3** Extra explanation of clustering algorithms

*K-means/K-medoids Clustering*

The participants were classified into distinct groups based on dietary consumption patterns, employing K-means clustering. The algorithm iteratively partitions the data into a pre-specified number of clusters, k, minimising the total within-cluster sum of squares (WCSS), which measures the total distance between each data point and its assigned cluster centroid. The k centroids are selected randomly. Each data point is assigned to the closest centroid. The new centroid for each cluster is calculated based on the mean points in each cluster. This is repeated until the clusters no longer change or the maximum number of iterations is reached [3].

For K-medoids clustering, the participants were classified into distinct groups based on dietary consumption patterns. The K-medoids algorithm functions analogously to the K-means algorithm. The distinction between the algorithms lies in identifying the cluster centre. K-means uses the mean point, whereas K-medoids utilises an actual point within the cluster [3].

Multiple models were generated with k ranging from 1 to 10 using the *fviz_nbclust* function to determine the optimal number of clusters (k). The WCSS values for each model were plotted to create an elbow plot, where the "elbow point" was defined as the optimal number of clusters. Together with the cluster size balance, the Silhouette Index, and the range of cluster numbers previously identified in the literature [4], this established the optimal number. The final K-means and K-medoids clustering was conducted using the *kmeans* and *pam* functions with the optimal cluster number, respectively. The algorithm was executed multiple times with different random initialisations of centroids to ensure stability.

*Hierarchical Clustering*

Agglomerative hierarchical clustering divided participants into distinct groups based on dietary consumption. The algorithm starts by treating each data point as its own cluster and then iteratively merges the closest clusters based on a chosen linkage method and distance measure. As the clusters merge, the distance between them is recalculated at each iteration. This process continues until all data points are in one cluster [3].

Four linkage methods were initially employed for the clustering analysis, namely average, single, complete and Ward's linkage. Subsequently, the agglomerative coefficients of the linkage methods selected Ward’s method as the most robust linkage method, as it yielded the strongest clustering. The agglomerative coefficients and the optimal cluster numbers were estimated using the R functions *agnes* and *NbClust*, respectively. Hierarchical clustering was conducted using Ward's method and the *hclust* function. The clustering results were presented in a dendrogram, a tree-like figure illustration depicting the hierarchical relationship between clusters.

*Density-based Clustering*

Clusters are areas of high point density next to one other in the data space, with low point density regions separating them. If the two points are density-reachable from one another, they are allocated to the same cluster. Density-based clustering techniques have the advantage of not requiring prior knowledge of determining the number of clusters k beforehand, discovering clusters of any shape, handling different noise levels, and not assuming parametric distributions. The DSBCAN model is the most popular one. The key parameters for DBSCAN are the neighbourhood radius, denoted by ε and the minimum number of points in a cluster, denoted by MinPts [5].

The initial value for MinPts was set to twice the number of features, according to a rule of thumb. K-distance graphs were constructed for selecting appropriate ε values, based on the "elbow" point. Multiple combinations of ε and MinPts were evaluated to examine the resilience of DBSCAN for the dataset. The K-distance graphs showed clear elbow points, suggesting suitable candidate ε values. However, despite testing multiple combinations of ε and MinPts, DBSCAN consistently resulted in a single cluster encompassing most data points. Therefore, it was impossible to compare DBSCAN with the other clustering methods, and DBSCAN was excluded from the analysis.

The Silhouette Index quantifies the degree to which a point is similar to its cluster (cohesion) compared to other clusters (separation). The value is between -1 and 1, with a high Silhouette Index value (1) indicating that the item is poorly matched to surrounding clusters and highly matched to its cluster [6]. The DB index is the ratio of the within-cluster dispersion to the between-cluster separation. The optimal clustering algorithm has the lowest DB Index [7]. The Dunn Index is the ratio of the shortest distance between observations not in the same cluster to the highest intra-cluster distance. The Dunn Index, which ranges from zero to infinity, is recommended to be maximised [8]. The CH Index is a metric that compares an object's cohesiveness (i.e. its similarity to its cluster) to its separation from other clusters. The ratio of the within-cluster variation to the between-cluster variance forms the foundation of the CH index. Higher values indicate better clustering quality [9].

# **Methods S4** Extra explanation of classification algorithms

*Decision Tree*

A DT is a non-parametric method which does not have distributional assumptions. It resembles a flowchart with internal nodes representing an attribute test, branches representing test results, and leaf nodes (terminal nodes) storing class labels. An attribute value test can divide the source set into subsets, thereby facilitating tree construction. This procedure, designated as recursive partitioning, is conducted recursively on each derived subset. The recursion ends when splitting no longer enhances the predictive accuracy or when the subset at a node exhibits homogeneity in the value of the target variable [10].

A DT model was constructed using the *train* function from the caret package, with the method set to "rpart". A grid search was conducted to evaluate the impact of varying values for the complexity parameter (CP). Based on the highest accuracy, the optimal CP value was then used for constructing the final model and a decision tree plot, utilising the *rpart* and *rpart.plot* functions, respectively. Subsequently, the final model was employed to make predictions on the test set and to evaluate the model.

*Random Forest*

A RF is an ensemble of trees, each dependent on a set of random factors. These ensembles or groups of decision trees are frequently grown by generating random vectors that control the development of each tree within the ensemble [11]. It utilises the majority voting classifier to determine the final outcome. The overfitting issue that the single DT creates is lessened by creating numerous decision trees in parallel for a given dataset [12]. Consequently, RF learning models that employ multiple decision trees are more accurate than those utilising a single DT [13]. The RF algorithm generates a set of decision trees with controlled variance by combining random feature selection [14] with bootstrap aggregation (bagging) [15].

An RF model was constructed using the *train* function from the caret package with the method set to "rf". The hyperparameter ntree (number of trees) was set to 500, and the nodesize (minimum size of terminal nodes) was set to the default. A grid search was conducted on the training dataset to evaluate different values for the number of variables used in each tree (mtry). The final model was selected based on the combination that yielded the highest accuracy. This model was tested and evaluated on the test dataset.

*Naïve Bayes*

NB classification is a straightforward probabilistic classification technique based on Bayes' theorem and predicated on the idea that features are independent. The NB method is referred to as "Naïve" due to its underlying assumption that the presence of a specific characteristic is independent of the presence of other features [16].

A basic NB model was constructed using the *train* function from the caret package, with the method set to "naive_bayes" from the e1071 package. Hyperparameter tuning was done on the usekernel hyperparameter and the Laplace smoothing hyperparameter. A grid search was conducted on the training dataset to evaluate all possible combinations of the hyperparameters. The final model was selected based on the combination that yielded the highest accuracy. The final model was then used to predict the test dataset.

*Support Vector Machine (SVM)*

To classify data points, SVM seeks to maximise the margin width of the line that separates the classes. Only the points closest to the line, known as support vectors, have the final say in determining the position of the separating line and the width of the margin. Even when the margin width is minimal, the separating line could result in a margin violation by a sample, which could occur if the sample falls within the margin or on the incorrect side of the line. However, allowing a few margin violations could improve the classification. The trade-off between margin width and misclassification is balanced by the hyperparameter C, usually chosen using cross-validation [17].

A basic SVM model was constructed using the *train* function from the caret package, with the radial kernel method set to "svmRadial" due to the presence of non-linear separable data. Based on the output of this basic SVM model, hyperparameter tuning was conducted on the C hyperparameter. Given the selection of the radial kernel, the hyperparameter sigma must be tuned to balance optimal performance and computational efficiency. A grid search was conducted to identify the optimal combination of hyperparameters, with the final model selected based on its accuracy. Subsequently, the model was employed to predict the test dataset.

*K-nearest neighbour (KNN)*

The KNN classification technique is a non-parametric ML model, which means that it does not make any assumptions about the shape of the class boundary. It identifies the K nearest neighbours in the training dataset relative to a new data point. The label assigned to the new data point is then that of the predominant class among its neighbours. A distance measure calculates the distance between a new data point and all the other data points to find the nearest neighbours [17, 18]. Besides, the user specifies the number of K neighbours used. Smaller k values can lead to overfitting, whereas larger k values could lead to underfitting [17].

The Euclidean distance metric determined the distance between a given data point and its nearest neighbours within the training dataset. The *train* function from the caret package, with the method set to "knn", was employed to identify the optimal value of K neighbours. The final model, with the optimal value of K neighbours, was then selected based on the highest accuracy. Subsequently, the final model predicted the test dataset.

*XgBoost*

XgBoost is an efficient and widely used gradient boosting library. Gradient boosting is an ensemble method that shares similarities with RF. Nevertheless, there are significant discrepancies in the construction and combination of trees. In contrast to the RF algorithm, gradient boosting employs a boosting technique rather than the bagging approach. This involves the construction of a robust model by combining a weak model with several additional weak models, intending to optimise a loss function via gradient descent (for XgBoost Newton descent). Subsequent trees rectify the shortcomings of those trained initially, thereby promoting a reduction in bias or underfitting. Consequently, the residual errors of the preceding model are used to optimise the subsequent model. The final model is derived from a weighted aggregation of all the tree forecasts [19, 20].

An XgBoost model is only capable of handling numeric data. Therefore, the categorical variables were encoded into zeros and ones using one-hot encoding. Initially, a default model was constructed using the *xgboost* function. Due to the high number of hyperparameters and running time, two rounds of hyperparameter tuning were performed. The tuning models were constructed using the *train* function from the caret package with the method set to "xgbTree". A grid search was then created to try different combinations of hyperparameters. The best model was chosen based on the highest accuracy. The hyperparameter values of the best model were then used in the subsequent model. Finally, the optimal hyperparameter values from the two rounds were employed in the final model, which was implemented via the *xgboost* function. The final model was then tested and evaluated on the test data.

# **References**

1. van Rossum C, Sanderman-Nawijn E, Brants H, Dinnissen C, Jansen-van der Vliet M, Beukers M, et al. The diet of the Dutch. Results of the Dutch National Food Consumption Survey 2019- 2021 on food consumption and evaluation with dietary guidelines. Rijksinstituut voor Volksgezondheid en Milieu RIVM; 2023 2023-10-31. Report No.: RIVM rapport 2022-0190.

2. Forman M (1999) Nutritional Epidemiology. Am J Clin Nutr 69:1020-1020. <https://doi.org/10.1093/ajcn/69.5.1020>

3. Murphy K, López-Pernas S, Saqr M (2024) Dissimilarity-Based Cluster Analysis of Educational Data: A Comparative Tutorial Using R. In: Saqr M, López-Pernas S, editors. Learning Analytics Methods and Tutorials: A Practical Guide Using R. Springer Nature Switzerland, Cham. p. 231-283.

4. Newby PK, Tucker KL (2004) Empirically Derived Eating Patterns Using Factor or Cluster Analysis: A Review. Nutr Rev 62(5):177-203. <https://doi.org/10.1111/j.1753-4887.2004.tb00040.x>

5. Hahsler M, Piekenbrock M, Doran D (2019) dbscan: Fast Density-Based Clustering with R. J Stat Softw 91(1):1 - 30. <https://doi.org/10.18637/jss.v091.i01>

6. Rousseeuw PJ (1987) Silhouettes: A graphical aid to the interpretation and validation of cluster analysis. J Comput Appl Math 20:53-65. <https://doi.org/10.1016/0377-0427(87)90125-7>

7. Davies DL, Bouldin DW (1979) A Cluster Separation Measure. IEEE Trans Pattern Anal Mach Intell PAMI-1(2):224-227. <https://doi.org/10.1109/TPAMI.1979.4766909>

8. Dunn† JC (1974) Well-Separated Clusters and Optimal Fuzzy Partitions. J Cybern 4(1):95-104. <https://doi.org/10.1080/01969727408546059>

9. Caliński T, Harabasz J (1974) A dendrite method for cluster analysis. Commun Stat 3(1):1-27. <https://doi.org/10.1080/03610927408827101>

10. Song YY, Lu Y (2015) Decision tree methods: applications for classification and prediction. Shanghai Arch Psychiatry 27(2):130-135. <https://doi.org/10.11919/j.issn.1002-0829.215044>

11. Breiman L (2001) Random Forests. Mach Learn 45(1):5-32. <https://doi.org/10.1023/A:1010933404324>

12. Sarker IH (2021) Machine Learning: Algorithms, Real-World Applications and Research Directions. SN COMPUT SCI 2(3):160. <https://doi.org/10.1007/s42979-021-00592-x>

13. Sarker IH, Kayes ASM, Watters P (2019) Effectiveness analysis of machine learning classification models for predicting personalized context-aware smartphone usage. J Big Data 6(1):57. <https://doi.org/10.1186/s40537-019-0219-y>

14. Ho TK (1998) The random subspace method for constructing decision forests. IEEE Trans Pattern Anal Mach Intell 20(8):832-844. <https://doi.org/10.1109/34.709601>

15. Breiman L (1996) Bagging predictors. Mach Learn 24(2):123-140. <https://doi.org/10.1007/BF00058655>

16. Zhang Z (2016) Naïve Bayes classification in R. Ann Transl Med 4(12):241. <https://doi.org/10.21037/atm.2016.03.38>

17. Bzdok D, Krzywinski M, Altman N (2018) Machine learning: supervised methods. Nat Methods 15(1):5-6. <https://doi.org/10.1038/nmeth.4551>

18. Uddin S, Haque I, Lu H, Moni MA, Gide E (2022) Comparative performance analysis of K-nearest neighbour (KNN) algorithm and its different variants for disease prediction. Sci Rep 12(1):6256. <https://doi.org/10.1038/s41598-022-10358-x>

19. Chen T, Guestrin C, editors. XGBoost: A scalable tree boosting system. Proceedings of the ACM SIGKDD International Conference on Knowledge Discovery and Data Mining; 2016; San Fransisco: Association for Computing Machinery.

20. Natekin A, Knoll A (2013) Gradient Boosting Machines, A Tutorial. Front Neurorobotics 7:21. <https://doi.org/10.3389/fnbot.2013.00021>

21. Yazici İ, Shayea I, Din J (2023) A survey of applications of artificial intelligence and machine learning in future mobile networks-enabled systems. Eng Sci Technol Int J 44:101455. <https://doi.org/10.1016/j.jestch.2023.101455>

22. Jin X, Han J (2010) K-Medoids Clustering. In: Sammut C, Webb GI, editors. Encyclopedia of Machine Learning. Springer US, Boston, MA. p. 564-565.

23. Kriegel H-P, Kröger P, Sander J, Zimek A (2011) Density-based clustering. WIREs Data Mining Knowl Discov 1(3):231-240. <https://doi.org/10.1002/widm.30>

24. Shetty P, Singh S (2021) Hierarchical Clustering: A Survey. Int J Appl Res 7(4):178-181. <https://doi.org/10.22271/allresearch.2021.v7.i4c.8484>

25. Bodnar LM, Kirkpatrick SI, Naimi AI (2022) Machine learning can improve the development of evidence-based dietary guidelines. Public Health Nutr 25(9):2566-2569. <https://doi.org/10.1017/s1368980022001392>
